# Effective Information Extraction Framework for Heterogeneous Clinical Reports Using Online Machine Learning and Controlled Vocabularies

**DOI:** 10.2196/medinform.7235

**Published:** 2017-05-09

**Authors:** Shuai Zheng, James J Lu, Nima Ghasemzadeh, Salim S Hayek, Arshed A Quyyumi, Fusheng Wang

**Affiliations:** ^1^ Department of Biomedical Informatics Emory University Atlanta, GA United States; ^2^ Department of Mathematics and Computer Science Emory University Atlanta, GA United States; ^3^ Division of Cardiology Emory School of Medicine Emory University Atlanta, GA United States; ^4^ Department of Biomedical Informatics Stony Brook University Stony Brook, NY United States

**Keywords:** information extraction, natural language processing, controlled vocabulary, electronic medical records

## Abstract

**Background:**

Extracting structured data from narrated medical reports is challenged by the complexity of heterogeneous structures and vocabularies and often requires significant manual effort. Traditional machine-based approaches lack the capability to take user feedbacks for improving the extraction algorithm in real time.

**Objective:**

Our goal was to provide a generic information extraction framework that can support diverse clinical reports and enables a dynamic interaction between a human and a machine that produces highly accurate results.

**Methods:**

A clinical information extraction system IDEAL-X has been built on top of online machine learning. It processes one document at a time, and user interactions are recorded as feedbacks to update the learning model in real time. The updated model is used to predict values for extraction in subsequent documents. Once prediction accuracy reaches a user-acceptable threshold, the remaining documents may be batch processed. A customizable controlled vocabulary may be used to support extraction.

**Results:**

Three datasets were used for experiments based on report styles: 100 cardiac catheterization procedure reports, 100 coronary angiographic reports, and 100 integrated reports—each combines history and physical report, discharge summary, outpatient clinic notes, outpatient clinic letter, and inpatient discharge medication report. Data extraction was performed by 3 methods: online machine learning, controlled vocabularies, and a combination of these. The system delivers results with F1 scores greater than 95%.

**Conclusions:**

IDEAL-X adopts a unique online machine learning–based approach combined with controlled vocabularies to support data extraction for clinical reports. The system can quickly learn and improve, thus it is highly adaptable.

## Introduction

While immense efforts have been made to enable structured data model for electronic medical record (EMR), a large amount of medical data remain in free-form narrative text, and useful data from individual patients are usually distributed across multiple reports of heterogeneous structures and vocabularies. This poses major challenges to traditional information extraction systems, as either costly training datasets or manually crafted rules have to be prepared. These approaches also lack the capability of taking user feedbacks, to adapt and improve the extraction algorithm in real time.

Our goal is to provide a generic information extraction framework that is adaptable to diverse clinical reports, enables a dynamic interaction between a human and a machine, and produces highly accurate results with minimal human effort. We have developed a system, Information and Data Extraction using Adaptive Online Learning (IDEAL-X), to support adaptive information extraction from diverse clinical reports with heterogeneous structures and vocabularies. The system is built on top of online machine learning and customizable controlled vocabularies. A *demo video* can be found on YouTube [1].

**Figure 1:**
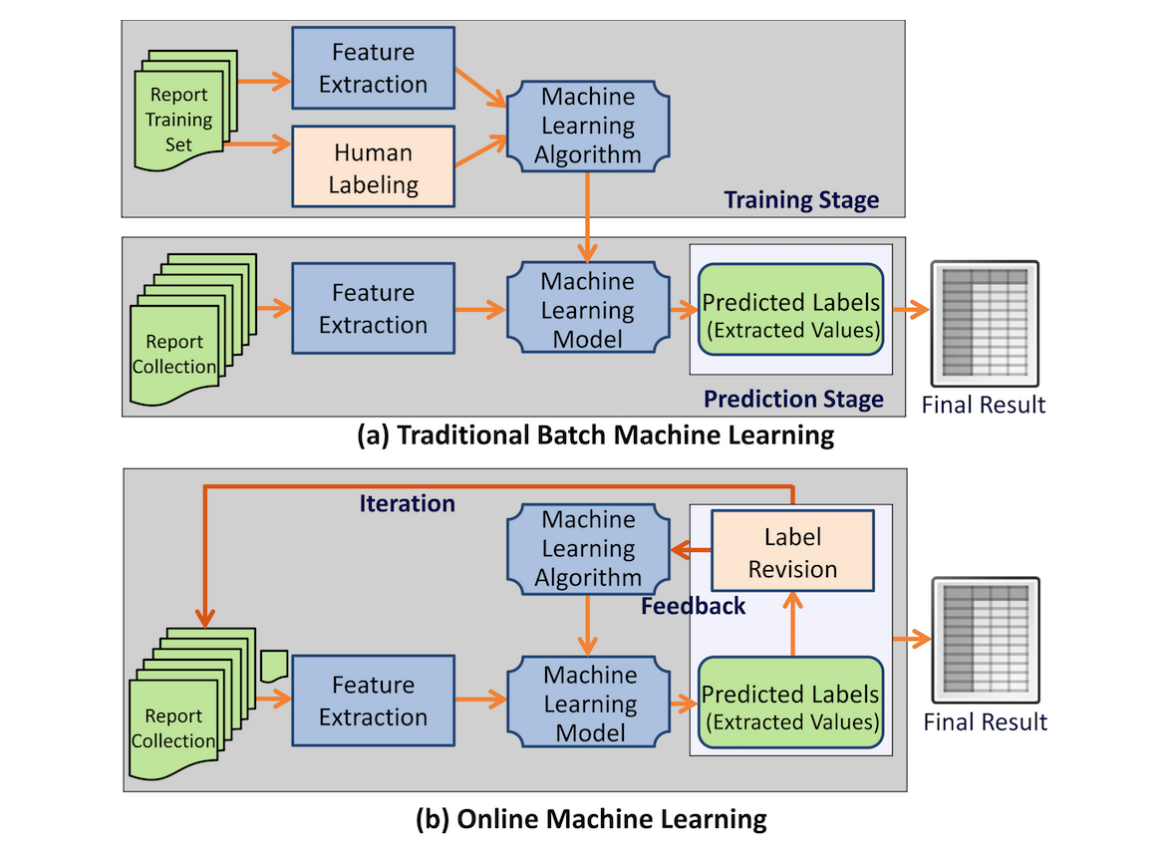
Online machine learning versus batch learning. (a) Batch machine learning workflow; (b) Online machine learning workflow.

IDEAL-X uses online machine learning–based approach [2-4] for information extraction. Traditional machine learning algorithms take a two-stage approach: batch training based on an annotated training dataset, and batch prediction for future datasets based on the model generated from stage one (Figure 1). In contrast, online machine learning algorithms [2,3] take an iterative approach (Figure 1). It learns one document at a time, and predicts values to be extracted for the next one. Learning occurs from revisions made by the user, and the updated model is applied to prediction for subsequent documents. Once the model achieves a satisfactory accuracy, the remaining documents may be processed in batch. Online machine learning not only significantly reduces human’s effort for annotation but also provides the mechanism for collecting feedback from human-machine interaction to improve the system’s model continuously.

Besides online machine learning, IDEAL-X allows for customizable controlled vocabularies to support data extraction from clinical reports, where a vocabulary enumerates the possible values that can be extracted for a given attribute. (The X in IDEAL-X represents the controlled vocabulary plug-in.) The use of online machine learning and controlled vocabularies is not mutually exclusive; they are complementary, which provide the user with a variety of modes for working with IDEAL-X.

## Background

### Related Work

A number of research efforts have been made in different fields of medical information extraction. Successful systems include caTIES [5], MedEx [6], MedLEE [7], cTAKES [8], MetaMap [9], HITEx [10], and so on. These methods either take a rule-based approach, a traditional machine learning–based approach, or a combination of both.

Different online learning algorithms have been studied and developed for classification tasks [11], but their direct application to information extraction has not been studied. Especially in the clinical environment, the effectiveness of these algorithms is yet to be examined. Several pioneering projects have used learning processes that involve user interaction and certain elements of IDEAL-X. I^2^E^2^ is an early rule-based interactive information extraction system [12]. It is limited by its restriction to a predefined feature set. Amilcare [13,14] is adaptable to different domains. Each domain requires an initial training that can be retrained on the basis of the user’s revision. Its algorithm (LP)^2^ is able to generalize and induce symbolic rules. RapTAT [15] is most similar to IDEAL-X in its goals. It preannotates text interactively to accelerate the annotation process. It uses a multinominal naïve Baysian algorithm for classification but does not appear to use contextual information beyond previously found values in its search process. This may limit its ability to extract certain value types.

Different from online machine learning but related is active learning [16,17], it assumes the ability to retrieve labels for the most informative data points while involving the users in the annotation process. DUALIST [18] allows users to select system-populated rules for feature annotation to support information extraction. Other example applications in health care informatics include word sense disambiguation [19] and phenotyping [20]. Active learning usually requires comprehending the entire corpus in order to pick the most useful data point. However, in a clinical environment, data arrive in a steaming fashion over time that limits our ability to choose data points. Hence, an online learning approach is more suitable.

IDEAL-X adopts the Hidden Markov Model for its compatibility with online learning, and for its efficiency and scalability. We will also describe a broader set of contextual information used by the learning algorithm to facilitate extraction of values of all types.

### Heterogeneous Clinical Reports

A patient’s electronic medical record could come with a variety of medical reports. Data in these reports provide critical information that can be used to improve clinical diagnosis and support biomedical research. For example, the Emory University Cardiovascular Biobank [21] collects records of patients with potential or confirmed coronary artery diseases undergoing cardiac catheterization, and aims to combine extracted data elements from multiple reports to identity patients for research. Report types include history and physical report, discharge summary, outpatient clinic note, outpatient clinic letter, coronary angiogram report, cardiac catheterization procedure report, echocardiogram report, inpatient report, and discharge medication lists.

**Figure 2:**
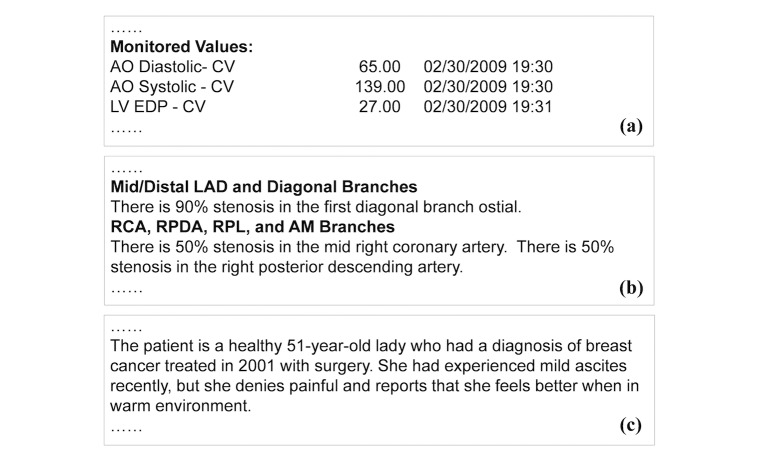
Example snippets of different report forms. (a) Semistructured report; (b) Template based narration; and (c) Complex narration.

We classify clinical reports into 3 forms: semistructured data, templatebased narration, and complex narration. Semistructured data represent data elements in the form of attribute and value pairs (Figure 2). Reports in this form have simple structures, making data extraction relatively straightforward. Template-based narration is a very common report form. The narrative style, including sentence patterns and vocabularies, follow consistent templates and expressions (Figure 2). Extracting information from this type of text (eg, “right posterior descending artery”) require major linguistics expertise, to either formulate extraction rules or to annotate training data. Complex narration is essentially free-form text. It can be irregular, personal, and idiomatic (Figure 2). Most medical reporting systems still allow for (and thus encourage) such a style. It is the most difficult form to interpret and process by NLP algorithms. Nevertheless, certain type of information such as diseases and medications has finite vocabulary that could be used to support data extraction.

## Methods

### Overview

The interface and workflow conform to traditional annotation systems: a user browses an input document from the input document collection and fills out an output form. On loading each document, the system attempts to fill the output form automatically with its data extraction engine. Then, a user can review and revise incorrect answers. The system then updates its data extraction model automatically based on the user’s feedbacks. Optionally, the user may provide a customized controlled vocabulary to further support data extraction and answer normalization. Pretraining with manually annotated data is not required, as the prediction model behind the data extraction engine can be established incrementally through online learning, customizing controlled vocabularies, or a combination of the two.

The system can operate in two modes: (1) interactive: through online learning, the system predicts values to be extracted for each report, and the user verifies or corrects the predicted values; and (2) batch: batch predicting for all unprocessed documents once the accrued accuracy is sufficient for users. Whereas interactive mode uses online machine learning to build the learning model incrementally, batch mode runs the same as the prediction phase of batch machine learning.

#### System Interface and User Operations

##### System Interface

**Figure 3:**
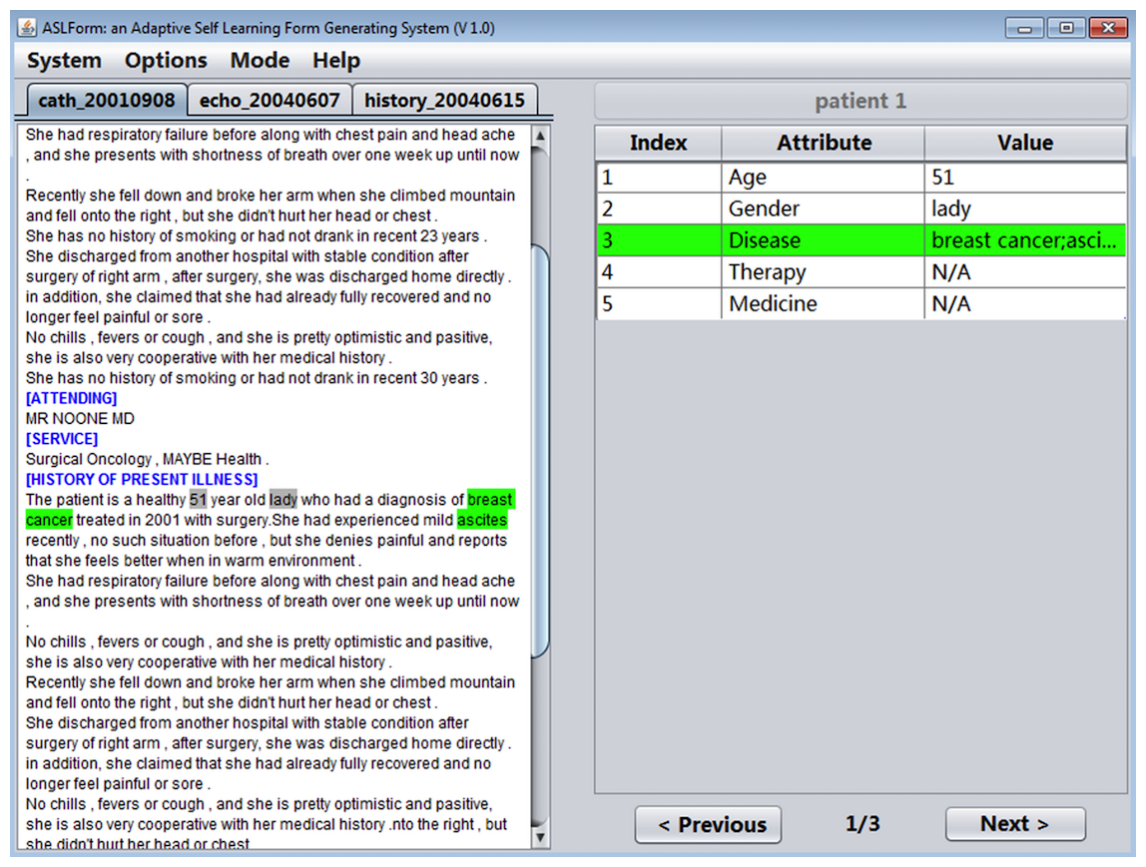
An example screenshot of IDEAL-X’s interface.

IDEAL-X provides a GUI with two main panels: a menu and navigation buttons (Figure 3). The left panel is for browsing an input report, and the right panel is the output table with predicted values of each data element in the report. The menu provides options for defining the data elements to be extracted, specifying input reports, among others.

##### Definition of Data Elements for Extraction

The system provides a wizard for constructing the metadata of the output form. The user builds the form by specifying a list of data elements and their constraints. An example is the data element “Heart Rate,” which is constrained to be a numerical value between 0 and 200. Other constraints include sections of the report that may contain the values. However, except for the names of the data elements, specifying constraints are optional, as these can be learned by the system.

##### Data Extraction Workflow

The user will first select a collection of input reports to be extracted from a local folder. By default, the system runs in an interactive mode, and one report will be loaded at a time on the left display panel. The user can make manual annotations by highlighting the correct value in the report text. Clicking the corresponding data field in the table assigns the value to the data element. If the system has prefilled the field of a data element with a predicted value, the user can provide feedback by fixing incorrect values. As the user navigates to the next document, the system compares the prefilled and the final values for the most recently processed document. Values that are unchanged or filled in by users are taken as positive instances, and values that have been revised are taken as negative instances. Both instances are incorporated into the online learning algorithm to be used by the data extraction for subsequent documents. By iterating through this process, the amount of information that the system is able to correctly prefill grows over time. Note that manual revision in this context is different from traditional human labeling. It is only necessary if there is a wrong prediction, thus humans’ effort can be significantly saved. Once the decision model reaches an acceptable level of accuracy, the user has the option to switch to batch mode to complete extraction for the remaining documents. If a patient has multiple reports, the text input panel displays each report with a separate tab. Data extracted from all the reports are aggregated in the output.

##### Customization of Controlled Vocabularies

IDEAL-X also provides an interface for the user to customize a controlled vocabulary that can be used by the system for data extraction. The controlled vocabulary contains both terminology and structural properties. The terminology includes lists of values and their normalization mappings. For example, Disease terminology includes “Diabetes Mellitus” with variations “DM” and “Diabetes.” It also defines inductions. For example, taking “Insulin” or “Metformin” indicates having Diabetes Mellitus. Structural properties provide positive and negative contextual information for giving terms. For example, to extract medications taken by patients, the “Allergies” section is a negative context and medicine names in the section will be skipped. Structural properties may also contain disambiguation terms that may further improve the precision of extraction. A simple example is that “intolerant” is a negative indicator for identifying “statin” as “statin intolerant” refers to different a concept. Controlled vocabularies can be a powerful tool to support data extraction: it can be used to locate sentences and chunks of possible values, and to perform normalization for extracted values, discussed in the next section.

##### The Data Extraction Engine

**Figure 4:**
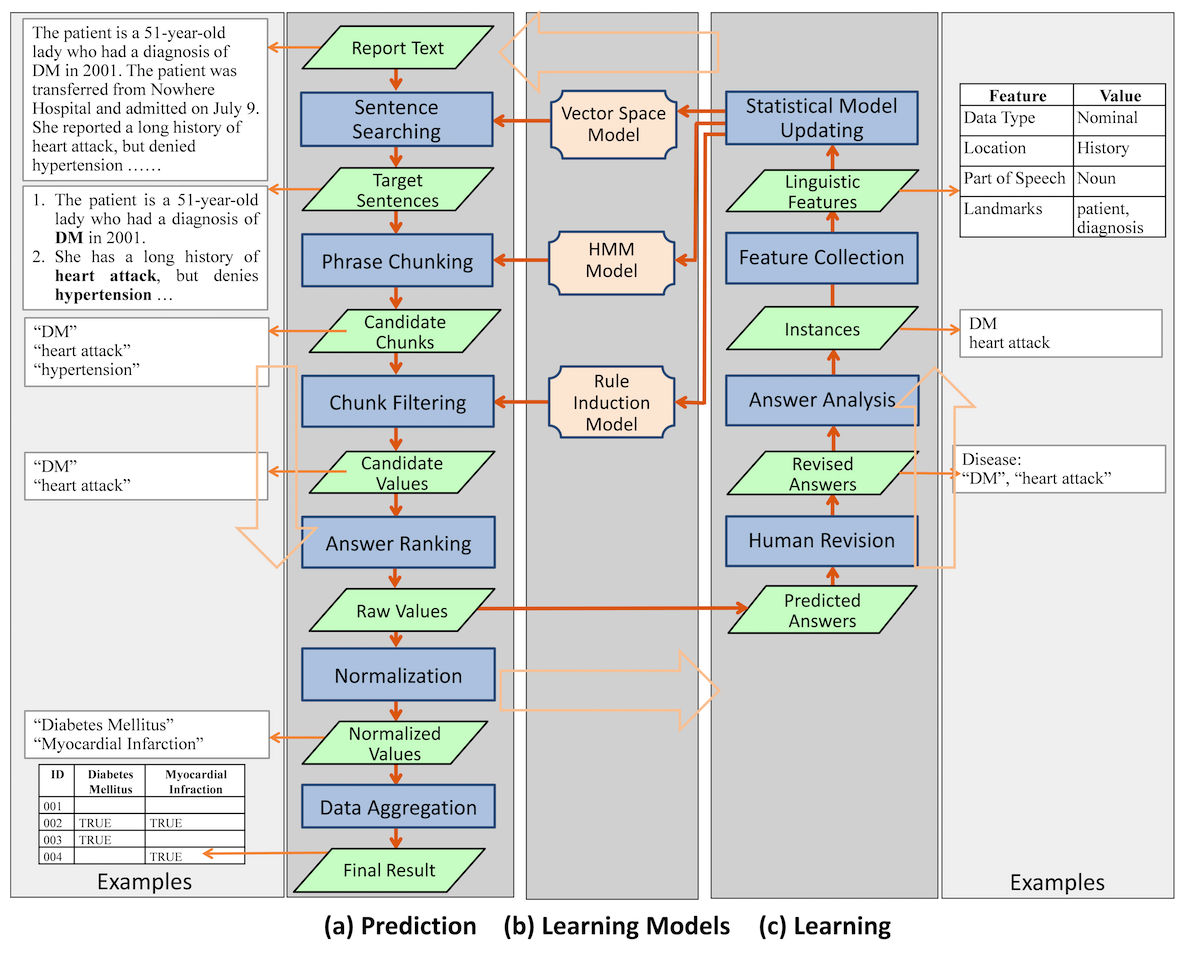
Overview of System Workflow.

While the user interacts with IDEAL-X interface, the data extraction engine works transparently in the background. The engine has 3 major components: answer prediction, learning, and the learning model that the online learning process continuously updates (Figure 4). The system combines statistical and machine learning–based approaches with controlled vocabularies for effective data extraction.

#### Document Preprocessing

When a report is loaded, the text is first parsed into an in-memory hierarchical tree consisting of 4 layers: section, paragraph, sentence, and token. Apache OpenNLP [22] is used to support the parsing with its Sentence Detector, Tokenizer, and Part-of-Speech Tagger. A reverse index of tokens is created to support efficient keywords-based search. The index is used to find locations (eg, sections, paragraphs, sentences, and phrases) of a token, as well as its properties such as part of speech and data type. For example, given the token “DM,” the system can quickly identify the section (eg, “History”) and the containing sentences. Such token search is frequently performed in answer prediction, and the in-memory index structures enable high efficiency for such operations.

#### Answer Prediction

**Figure 5:**
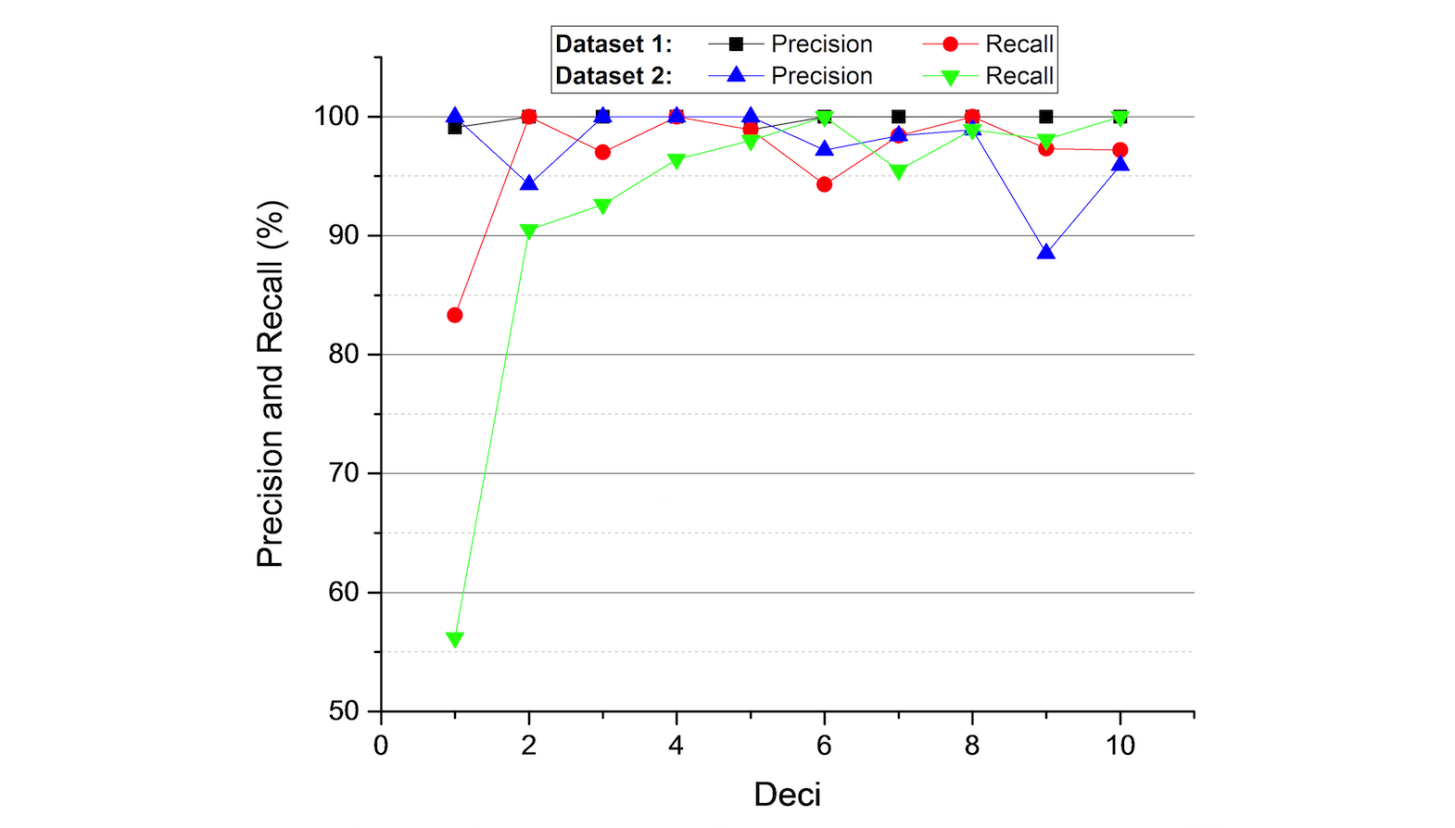
Precision and recall changes over processed records.

Predicting the value of each data element involves the following steps: (1) *Identifying target sentences* that are likely to contain the answer; (2) *Identifying candidate chunks* in the sentences; (3) *Filtering the chunks* to generate candidate values; (4) *Ranking candidate values* to generate (raw) values; (5) *Normalizing values*; and (6) *Aggregating values* from multiple reports. The workflow is shown in Figure 5.

##### Identifying Target Sentences

Through online learning, the system accrues keywords from past answers (*answer keywords*) along with cooccurring words in the corresponding sentences (*contextual words*). For example, given the answer keywords “diabetes” and “hypertension” in the sentence “The patient reports history of diabetes and hypertension,” contextual words are “patient,” “report,” and “history.” Such answer keywords and contextual words combined with customized vocabularies can be utilized to identify sentences that are likely to contain answers with the following methods:

First, similarity-based search using the vector space model [23]. Given a collection of contextual words and their frequencies, the system computes the similarity against sentences in the document [23]. Sentences with high similarities are selected. For example, most sentences about “disease” contain “diagnosis” and “history.” The past contextual keywords and their frequency weights are represented and maintained through a learning model discussed later in “Learning” section.

Second, answer keyword matching search. The answer keywords, combined with relevant user customized vocabularies, are also used to identify target sentences with keyword matching. For example, to extract diseases, if a sentence contains the disease term “myocardial infarction” defined in the vocabulary, the sentence is selected as a target. In both approaches, sections to be searched or skipped are also considered to narrow the scope of searching.

##### Identifying Candidate Chunks

After target sentences are selected, the system identifies potential phrases in the sentences using 2 methods: Hidden Markov model (HMM) [24] and keyword-based search. The HMM represents target words and contextual words in a sentence with different states, and marks values to be extracted based on probability distributions learned from previously collected values and their sentences. The keyword-based search finds candidate chunks using keywords collected from past answers and the controlled vocabulary.

##### Filtering chunks

To filter candidate chunks, the system uses rule induction [14,25] to generate “If-Then” rules based on historical statistics. The following filtering criteria are used: (1) Part of speech (POS): This filters a phrase by its POS tag in the sentence. Simple example phrases are noun and verb phrases. (2) String pattern: This looks for chunks that match special string patterns. For example, the first characters of all tokens are capitalized. (3) Value domain: This eliminates numerical or enumerated values that fall outside a specified range of values. (4) Negation: Based on predefined built-in rules, this removes phrases governed by words that reverse the meaning of the answer [26]. For example, if a candidate chunk “cancer” is extracted from a sentence “the patient has no history of cancer,” “cancer” would not be included. (5) Certainty: Similar to negation filter, this detects and filters uncertain events or situations such as future plans, based on predefined rules. For example, a candidate chunk “radiation therapy” for treatment from a sentence “the patient is planned to take radiation therapy” should not be included. Whereas negation and certainty filtering is based on predefined rules, other filtering relies on real-time data statistics for filtering criteria.

##### Ranking Candidate Values

The system combines the scores of the selected sentences and chunks for ranking of candidate values. For a single-valued data element (eg, heart beat), the candidate value with the highest confidence score is selected. For a multi-valued data element (eg, medication), values with confidence scores above a threshold are selected. Based on this, each candidate value is either accepted or rejected.

##### Normalizing Values

This step normalizes extracted values through transformation, generalization, and induction rules given by the controlled vocabulary (Figure 4). For example, “DM” is transformed into “Diabetes Mellitus.” “Pindolol” is generalized to its hypernym “beta blocker.” The appearance of medication term “Metformin” (a drug for treating type 2 diabetes) in the text can infer the disease “Diabetes Mellitus.”

##### Aggregating Results

Data extracted from multiple reports of a patient will be aggregated into a single table. The aggregation process may normalize values and remove duplicates. For example, “lisinopril” and “captopril” are extracted from discharge summary and inpatient report, respectively, and they can be normalized as “ACE inhibitor.” If the same data element is extracted from multiple reports, deduplication is performed. The final output is in simple structural table form that can be exported conveniently to other applications such as Excel (Microsoft) or a database.

Note that controlled vocabularies can play important roles in the answer prediction process. They are used for identifying target sentences through keyword searching, identifying candidate chunks through keyword matching, and supporting normalization for extracted values.

#### Learning

IDEAL-X takes an online learning–based approach to incrementally build statistical models and make predictions (Figure 5). The 3 models used in IDEAL-X are all statistical based and can be continuously updated after each iteration.

System-predicted values automatically populate the output table, and the user advances to the next report with or without revision to these values. In both cases, the internal learning and prediction models of IDEAL-X are updated. For each instance, IDEAL-X collects and analyzes the following features: (1) Position: location of the answer in the text hierarchy; (2) Landmark: co-occurring contextual keywords in a sentence; (3) POS: parts of speech tag; (4) Value: the tokens of the answer; (5) String patterns: literal features such as capitalization and initial and special punctuation. These features are then used to update the 3 models.

In IDEAL-X, each data element such as attribute “disease” or “medicine,” has its own statistical model, and each new instance of a data element will update the corresponding model. There are 3 models to be updated: (1) Updating Space Vector Model: This model uses “Landmark” features of positive instances. The system updates frequencies of cooccurring contextual words, used as weights of the space vector [23]. (2) Updating HMM: HMM lists all words in a sentence as a sequence, in which an extracted value is marked as target value state and other words are recognized as irrelevant contextual states. Based on this sequence, the state transition probabilities and emission probabilities are recalculated [24]. (3) Updating rule induction model: Filtering rules are induced based on the coverage percentage [25]. Features such as POS, value domain and string patterns of both positive and negative instances are analyzed and their respective coverage percentages are modified. Once the coverage of a rule reaches a predefined threshold, the rule is triggered for filtering.

In an interactive mode, the above 4 steps repeat for each report, where the learning models are continuously updated and improved.

## Results

### Experimental Setup

#### Datasets

We used 3 datasets from 100 patients that were randomly sampled from a collection of about 5000 patients in the Emory Biobank database. Dataset 1 is a set of semistructured reports and contains 100 cardiac catheterization procedure reports. Dataset 2 is a set of template-based narration and contains 100 coronary angiographic reports. Dataset 3 is a set of complex narration and contains 315 reports, including history and physical report, discharge summary, outpatient clinic notes, outpatient clinic letter, and inpatient discharge medication report.

#### Ground Truth

The test datasets are independently hand-annotated by domain expert annotators, including physicians, physician trainees, and students trained by the Emory Clinical Cardiovascular Research Institute for Biobank data reporting. Each record is annotated by 2 different annotators. The interrater agreement scores (kappa) of these 3 datasets are .991, .986, and .835, respectively. An arbitrator—an independent cardiovascular disease researcher reconciles incompatible outputs of the system and the manual annotations to produce the final ground truth.

#### Evaluation Metrics

For validation, precision, recall, and F1 scores are used to estimate the effectiveness of extraction by comparing the system predicted results (before human revision) and the ground truth.

#### Experiment Settings

We aimed to evaluate the effectiveness of the system with respect to using online learning and controlled vocabularies and to understand their applicability to different report forms. By analyzing the report styles and vocabularies, we discovered that online learning will be more suitable for semistructured or template-based narration reports, and controlled vocabulary-guided data extraction would be more effective on complex narration with a finite vocabulary. Thus, we designed 3 experiments: (1) Online learning–based data extraction, where controlled vocabularies are not provided, based on Dataset 1 (semistructured) and Dataset 2 (template-based narration); (2) Controlled vocabularies-based data extraction, where online learning is not used, based on Dataset 3 (complex narration); and (3) Controlled vocabularies guided data extraction combined with online learning, based on Dataset 3.

### Performance Evaluation

#### Experiment 1: Online machine Learning–Based Data Extraction

This experiment was based on Datasets 1 and 2. The system starts in an interactive mode with an empty decision model without prior training. The defined data elements are summarized in Multimedia Appendix 1. The user processes one report at a time, and each system-predicted value (including empty values for the first few reports) before user revision was recorded for calculating precision and recall.

Results are summarized in Table 1 for the 2 datasets, respectively. Both test cases achieved high precision as semistructured and template-based text is most easy to handle. To study the learning rate of online learning, we divided records into 10 groups, and plotted precision and recall of every 10% of the records in datasets 1 and 2. We observed that in both tests, the system maintained high precision during the learning process. Although some variability exists due to new data pattern, the recall of both cases also improved steadily. Not surprisingly, the rate of learning for dataset 1 is much faster given its semistructure.

#### Experiment 2: Controlled Vocabularies-Guided Data Extraction

In this experiment, online learning was disabled and data extraction was performed in batches using controlled vocabulary. Diseases and medications were extracted from Dataset 3 (values to be extracted are shown in Multimedia Appendix 1). Customized controlled vocabularies, including terminology and structural properties, had been created independently by physicians through referring to domain knowledge resources and analyzing another development report dataset of 100 patients, disjoint from Dataset 3. Note that comparisons in this and the following experiments were at a clinical finding level between system-integrated-results and manual-annotation-integrated-results.

**Table 1:** Results of data extraction from semistructured reports (Dataset 1) and template-based narration (Dataset 2).

Dataset	Numbers of data elements	Number of values	Precision (%)	Recall (%)	F1 (%)
1	19	1272	99.8	96.5	98.1
2	16	728	97.2	93.2	95.2

**Table 2:** Results of controlled vocabularies-guided data extraction from complex narration (Dataset 3).

Type of data elements	Number of data elements	Number of round truth values	Precision (%)	Recall (%)	F1 (%)
Diseases	15	418	94.5	99.0	96.7
Medications	10	437	98.6	99.7	99.2
All	25	855	96.5	99.4	97.9

The results in Table 2 show that controlled vocabularies are highly effective for data extraction over complex narratives. Domain-specific data, for example, cardiology-related diseases and medications, have limited numbers of possible values (or domain values), and a carefully customized controlled vocabulary can achieve high extraction accuracy.

#### Experiment 3: Controlled Vocabularies-Guided Data Extraction Combined With Online machine Learning

In this experiment, we performed 2 tests to examine how efficient and effective the system learns when only terminology is available and structural properties need to be obtained from online learning. Test 1 was to generate the baseline for comparison, and Test 2 was to demonstrate the effectiveness of combining online machine learning and controlled vocabularies. Dataset 3 was used to extract all diseases and medications.

For Test 1, terminology was used and online machine learning is disabled, so the test was guided by controlled vocabulary without any structural properties. We note that comprehensive terminology contributes directly to high recall rate, which means that the system seldom misses values to be extracted. However, if structural properties are not included, compared with the result in Experiment 2, the precision is much lower. This highlights the value of positive and negative contexts in an extraction task.

For Test 2, both terminology and online machine learning were used. Online machine learning supports learning structural properties. To show how quickly the system learns, only the 38 reports associated with the first 10 patients were processed with interactive online learning. All remaining reports were processed in batch. Results in Table 3 show an overall precision of 94.9%, which demonstrates that online learning could quickly learn structural properties.

Typical errors in these 2 tests were associated with terminology and contextual information used in complex narrative scenarios. On one hand, the completeness of the terminology list, including terms and their synonyms, influences the recall rate directly. On the other hand, although coverage of terminologies could be maximized by a carefully engineered vocabulary, unwanted extractions arising from searches in the wrong section, undetected negations, and ambiguous use of terms can still lower the overall precision.

**Table 3:** Results of controlled vocabularies-guided data extraction combined with online learning.

Test	Controlled vocabulary	Online learning	Precision (%)	Recall (%)	F1 (%)
1	Terminology only	N/A	80.9	99.4	89.2
2	Terminology only	Applied to first 10 patients	94.9	99.4	97.1

## Discussion

### Principal Findings

IDEAL-X provides a generic data extraction framework that takes advantage of both online learning and controlled vocabularies. The 2 approaches complement each other and can also be combined. Online learning–based approach is highly effective for reports with underlying structural patterns such as semistructured or template-based narration style-based reports. Experiments with complex narrative reports indicate that the use of controlled vocabularies is highly effective for supporting extraction constrained by finite data domain. In addition, structural properties such as section-data associations can play an important role in improving the accuracy of extraction. However, in cases where controlled vocabularies are unavailable—extracting generic named entities for example, maintaining high accuracy is a challenge. This is an ongoing area of exploration that we will report in the future.

Machine learning is among major techniques for identifying candidate chunks. Besides HMM, we have also explored other classifiers such as Naive Bayes classifier and neural networks-based classifier. An ongoing project includes a systematic study of different classifiers and their combinations (including Conditional Random Field and Support Vector Machine [27]) for online machine learning–based data extraction.

To make it more flexible on using standard medical terminologies for customizing controlled vocabularies, an ongoing work is developing a tool that can easily search and import concepts from standard vocabularies such as ICDE-9, ICD-10, and SNOMED, from a local file or through NCBO BioPortal.

### Conclusions

Although there are natural language processing tools available for extracting information from clinical reports, the majority lack the capability to support interactive feedback from human users. An interactive, online approach allows the user to coach the system using knowledge specific to the given set of reports, which may include local reporting conventions and structures. Moreover, no advanced linguistics knowledge or programming skills are required of the users; the system maintains the ordinary workflow of manual annotation systems. We perform a systematic study on the effectiveness of the online learning–based method combining with controlled vocabularies for data extraction from reports with various structural patterns, and conclude that our method is highly effective. The framework is generic and the applicability is demonstrated with diverse report types. The software will be made freely available online [28].

## Appendix

Multimedia Appendix 1 Test cases.

## Abbreviations

EMR: electronic medical record
HMM: hidden markov model
NLP: natural language processing
POS: part of speech
