# Supplementary material for: Effective Information Extraction Framework for Heterogeneous Clinical Reports Using Online Machine Learning and Controlled Vocabularies
Source: JMIR Med Inform. 2017 May 9;5(2):e12. doi: 10.2196/medinform.7235 (PMC5442348; doi:10.2196/medinform.7235)
Supplement: Multimedia Appendix 1 [file medinform_v5i2e12_app1.pdf]

Table 5. Attributes for Cardiac Catheterization Procedure Reports (Dataset 1) Test Case

Aortic Diastolic (Ao) Pressure	Aortic Diastolic (Ao) Systolic Pressure	Aortic Diastolic (Ao) Mean Pressure
Left Ventricular End Diastolic Pressure (LVEDP)	Left Ventricular (LV) Systolic	Heparin Amount
Bivalirudin Amount	Abciximab Amount	Fentanyl Amount
Midazolam Amount	Nitroglycerin Amount	Acetylcholine Amount
Heparin Dosage	Bivalirudin Dosage	Abciximab Dosage
Fentanyl Dosage	Midazolam Dosage	Nitroglycerin Dosage
Acetylcholine Dosage		

Table 6. Attributes (Stenosis Values of) for Coronary Angiogram Reports (Dataset 2) Test Case

Stenosis value of:

Left Main Coronary Artery	First Diagonal Branches	Second Diagonal Branches
Proximal Circumflex Coronary Artery	Mid Circumflex Coronary Artery	Distal Circumflex Coronary Artery
Ramus	First Obtuse Marginal Branches	Second Obtuse Marginal Branches
Third Obtuse Marginal Branches	Proximal Right Coronary Artery	Mid Right Coronary Artery
Distal Right Coronary Artery	Proximal Circumflex Coronary Artery	Mid Circumflex Coronary Artery
Distal Circumflex Coronary Artery		

Table 7. Attributes for Complex Narration Data Extraction for Dataset 3 Test Case

a. Diseases:

**Name**	**Positive Case Amount**
Diabetes	**40**
Coronary Artery Bypass Grafting	**27**
Heart Transplant	**12**
Stroke	**11**
Peripheral Vascular Disease	**15**
Coronary Artery Disease	**74**
Asthma	**9**
Chronic Obstructive Pulmonary Disease	**14**
Percutaneous coronary intervention	**39**
Myocardial Infarction	**28**
Hypertension	**89**
Atrial Flutter	**0**

Alcohol	**20**
Heart Failure	**22**
Atrial Fibrillation	**18**

b. Medications:

**Name**	**Positive Case Amount**
Angiotensin II Receptor Blocker	**16**
Thiazides	**24**
Warfarin	**17**
Aspirin	**84**
Thienopyridine	**51**
Calcium Channel Blockers	**32**
Beta-Blockers	**70**
Statin	**79**
Loop Diuretics	**26**
Angiotensin-Converting-Enzyme Inhibitor	**38**
